# Supplementary material for: Rift Valley fever virus 78kDa envelope protein attenuates virus replication in macrophage-derived cell lines and viral virulence in mice
Source: PLoS Negl Trop Dis. 2021 Sep 13;15(9):e0009785. doi: 10.1371/journal.pntd.0009785 (PMC8460012; doi:10.1371/journal.pntd.0009785)
Supplement: Attachment — Submitted filename: Response to reviewers.docx [file pntd.0009785.s001.docx]

**Reviewer #1**: Overall the results presented are of high quality and make a significant contribution to the field.  However, much of the work was performed with MP-12 even though the authors have produced P78-High virus in a ZH501 background.  Thus, additional experiments using ZH501 P78-High (and P78-KO if possible) would strengthen the research.

**Major comments**

1) The authors should examine the replication of P78-High in mosquito cells.

Our response: To respond to the reviewer’s comments, we have tested growth kinetics of MP-12 and its variants in C6/36 mosquito cells (Fig. 3A) in the revised manuscript.

2) The authors should check replication kinetics of ZH501 P78-High (and P78-KO if possible) in at least Raw and THP-1 cells.  This experiment is important as it will determine if increased expression of P78 also suppresses viral replication in macrophages with a virulent strain of RVFV.

Our response: We have tested growth kinetics of ZH501 and ZH508-P78-High virus in Raw 264.7 and THP-1 cells, the latter of which were differentiated by PMA treatment. The data were shown in Fig. 3B in the revised manuscript. Because our results showed that MP-12 based P78-KO virus had increased virulence compared to MP-12 (Fig. 6 in original manuscript), there was a possibility that ZH501-based P78-KO virus might have increased virulence compared to parental ZH501. Because of this concern, we decided not to generate ZH501-based P78-KO virus.

3) Given the conflicting results with ZH548 (line 523-525), it is important for the authors to test their ZH501 mutant viruses in mice. However, use of 16-day old mice may not be appropriate. Rather testing in older immune competent mice with lower doses of virus would enable an evaluation of P78-High and P78KO virulence.

Our response: To respond to this comment, we added new data in Fig. 7 in the revised manuscript. We tested virulence of ZH501 and ZH501-P78-High virus in 5-week-old CD1 mice. The new result showed that ZH501-P78-High virus had attenuated virulence in adult mice. Although the reviewer suggested to test ZH501-P78-KO virus as well, we did not generate ZH501-P78-KO virus due to a concern of generating a more virulent virus than wild type RVFV as described above. Therefore, ZH501-P78-KO virus was not tested for its virulence this time.

**Minor comments:**
1) As the authors have already published on P78-KO virus (reference 15) it would be helpful if they could indicate the previous name of the virus one time in the manuscript.

Our response: We indicated the previous name in line 191.

2) Fig 1D: The ratio of p78 to Gn signal was discussed (lines 308-309) but is not shown in the figure.  Please add to the figure.

Our response: Our description about the ratio of P78 to Gn was not correct, as we could not directly compare the band intensities of Gn and P78, both of which were detected by different antibodies. We altered the description about the relative ratio of P78 to Gn in lines 330-333.

**Reviewer #2**: In this manuscript, the authors suggest a novel insight into cell tropism of Rift Valley fever virus, which may be regulated by the expression of P78 viral glycoprotein and its incorporation into the virions. This study also reemphasizes that efficient replication of RVFV in macrophages may be the key determinant of virulence in vivo. Although these findings are of interest and potentially provide insights into novel functions of GP78 in RVFV replication and pathogenesis, the study is fairly descriptive; therefore, the following critical issues should be addressed to improve this manuscript prior to publication.

1. Figure 3, to examine the impact of high expression of GP78 on RVFV growth in mosquito cells, this reviewer strongly recommends the authors to perform a growth kinetics study in C6/36 cells.

Our response: We have tested the growth kinetics of MP-12 and its variants in C6/36 mosquito cells. The data were shown in Fig. 3A in the revised manuscript.

2. Figures 4 and 5, the authors should perform the experiments with more appropriate cell lines such as THP-1, Huh7 (for Fig. 5), and C6/36 to discuss the role(s) of GP78 in pathogenesis and cell/host tropism.

Our response: To respond to the reviewer’s comments, we have tested the infectivity of MP-12 and its variants in C6/36 mosquito cells and PMA-treated THP-1 cells. The data were shown in Fig. 4 in the revised manuscript. Because the infectivity of the viruses in C6/36 and THP-1 were very low, high MOI infection was performed for these cell lines.

We also performed binding assays with Huh7, C6/36, and THP-1 cells. The results obtained using Huh7 and C6/36 cells were added in Fig. 5. Due to the low infectivity of the viruses in THP-1 cells, we detected very low amount of viral RNA after 1 h of incubation for virus binding. Because the copy number of viral RNA detected from the THP-1 samples were close to detection limit of our RT-qPCR system, we considered that reliability of the data was not sufficient for publication. Therefore, we did not show the results using THP-1 samples.

3. Figure 6, to define the role of GP78 in macrophage tropism and virus growth ability in vivo (blood, liver, and spleen), the authors should perform (i) immunohistochemistry to detect RVFV antigens in macrophages and other parenchymal cells and compare the numbers of RVFV-positive macrophages among wild-type, GP78-high, and GP78-KO mutants.

Our response: Although the suggested studies are meaningful for accessing the role of P78 in macrophage tropism and virus growth ability *in vivo*, we think that additional studies, including analyses of virus dissemination status, virus replication in various tissues, and analyses of host immune responses, etc., will be also necessary for fully addressing the reviewer’s comments. We consider these suggested studies as outside of the scope of the current study and are more appropriate to be performed in a separate study that aims to clarify the role of P78 in virus replication in various organs and virus cell tropisms in infected animals. Accordingly, we did not perform the suggested experiment in the present study.
